# A Genetic Interaction Network Model of a Complex Neurological Disease

**DOI:** 10.1111/gbb.12178

**Published:** 2014-10-27

**Authors:** Anna L. Tyler, Tracy C. McGarr, Barbara J. Beyer, Wayne N. Frankel, Gregory W. Carter

**Affiliations:** 1 The Jackson Laboratory, 600 Main St. Bar Harbor, ME, 04609, USA

## Abstract

Absence epilepsy (AE) is a complex, heritable disease characterized by a brief disruption of normal behavior and accompanying spike wave discharges (SWD) on the electroencephalogram. Only a handful of genes has been definitively associated with AE in humans and rodent models. Most studies suggest that genetic interactions play a large role in the etiology and severity of AE, but mapping and understanding their architecture remains a challenge, requiring new computational approaches. Here we use Combined Analysis of Pleiotropy and Epistasis (CAPE) to detect and interpret genetic interactions in a meta-population derived from three C3H x B6 strain crosses, each of which is fixed for a different SWD-causing mutation. Although each mutation causes SWD through a different molecular mechanism, the phenotypes caused by each mutation are exacerbated on the C3H genetic background compared with B6, suggesting common modifiers. By combining information across two phenotypic measures – SWD duration and frequency – CAPE revealed a large, directed genetic network consisting of suppressive and enhancing interactions between loci on 10 chromosomes. These results illustrate the power of CAPE in identifying novel modifier loci and interactions in a complex neurological disease, towards a more comprehensive view of its underlying genetic architecture.

## Introduction

Absence epilepsy (AE) is a form of idiopathic generalized epilepsy characterized by brief disruption of normal behavior concurrent with spike-wave discharges (SWD) on an electroencephalogram (EEG). In humans AE has a strong genetic component (Berkovic et al. 1998; R. H. Wallace et al. 2001; Hempelmann et al. 2006; Marini et al. 2003; Chen et al. 2003), and over the past several decades a number of genes, for example CACNA1H (Jouvenceau et al. 2001; Liang et al. 2006; Chen et al. 2003) and GABRG2, have been associated with this condition in large family studies (for reviews see Nicita et al. 2012; Lucarini et al. 2007; Crunelli and Leresche 2002).

Despite modest progress, the genetics of AE is still largely unknown. Human (Weber and Lerche 2008; Ottman 2005) and rodent (Frankel 2009; Rudolf et al. 2004; Powell et al. 2009) studies estimate that most epilepsies have complex inheritance patterns. One potential source for this complexity is an interacting, polygenic etiology underlying the disorder. Analysis of such a complex trait requires the use of novel analytical strategies that specifically address genetic interactions.

Here we investigate genetic interactions influencing SWD in mice using a novel algorithm called the Combined Analysis of Pleiotropy and Epistasis (CAPE) (Carter et al. 2012; Tyler et al. 2013). We combined three populations of mice, each with one mutation causing genetically complex SWD – *Gria4*, *Scn8a*, or *Gabrg2. Gria4* encodes one of four amino-3-hydroxy-5-methyl-4-isoxazolepropionic acid (AMPA) receptor subunits (Hollmann and Heinemann 1994). Spontaneous and engineered mutations of this gene in mice cause severely reduced protein levels and SWD (Beyer et al. 2008). Mutations in *Scn8a*, which encodes the alpha subunit of the voltage-gated sodium channel type VIII (Smith et al. 1998) also causes SWD in mice (Papale et al. 2009). Finally, *Gabrg2* encodes the gamma-aminobutyric acid (GABA) A receptor, subunit *γ* 2, and has been associated with AE and FS in humans (Wallace et al. 2001; Marini et al. 2003) and mice (Petrou 2010).

Mutations in each of these genes cause SWD with strain-dependent severity, suggesting multigenic modification of the phenotype. Specifically, each mutation causes more severe SWD in the C3H background than the B6J background (Papale et al. 2009; Oliva et al. 2014; Beyer et al. 2008; Tokuda et al. 2009; Frankel et al. 2009; Frankel et al., 2014;). These common strain effects suggest the possibility of common modifier(s) across multiple biochemical modalities and thus potential targets of broad therapeutic appeal.

Interval mapping focusing on the individual mutations has yielded significant quantitative trait loci (QTL), but no common modifiers across the three mutations, and no significant interactions (Oliva et al. 2014; Frankel et al. 2014; data not shown). To better analyze common modifiers across the three mutations, we combined the three crosses creating a “meta-cross,” and used CAPE to detect genetic interactions. CAPE increases power to detect genetic interactions and main effects by combining information from multiple phenotypes and systematically conditioning on all loci. The interactions are interpreted into directed influences of allelic suppression and enhancement, which aids in their interpretation and in specification of candidate genes.

## Methods

### Animal care

All animals were fed standard National Institutes of Health diet containing 6% fat and acidified water *ad libitum*, and maintained in a climate-controlled room with a 12 hour light on/off cycle. All animal procedures followed Association for Assessment and Accreditation of Laboratory Animal Care guidelines and were approved by institutional Animal Care and Use Committee.

### Mouse strains and genetic mapping

C57BL/6J (B6J) and C3HeB/FeJ (FeJ) inbred strains were obtained from The Jackson Laboratory production facility. The origins of *Gria4^spkw1^* (IAP insertion allele) *Gria4^tm1Dgen^* (null allele) and *Scn8a^8J^* (V929F allele) mutant mice were described previously (Beyer et al. 2008; Papale et al. 2009). B6J.129.*Gabrg2^tm1Spet^* (hereafter referred to as *Gabrg2^R43Q^* (Tan et al. 2007)) mice were obtained from Bionomics, Ltd (Thebarton, SA). Each mutation was backcrossed to B6J or FeJ strains for at least 10 generations, selecting for the mutation of origin by selective PCR genotyping using primers described in the original papers (Beyer et al. 2008; Papale et al. 2009). Three genetic mapping populations were used in this study. For *Scn8a*, an N_2_ backcross population was utilized, crossing a (B6J-*Scn8a^8J^* x FeJ)F_1_ hybrid to B6J to generate an N_2_ population comprising of 26 females and 15 males. For *Gabrg2*, (B6J.129-*Gabrg2^tm1Spet(R43Q)^* x FeJ)F_1_ hybrids were intercrossed, generating an F_2_ population comprising 9 females and 51 males. For *Gria4*, FeJ mice with the *Gria4^spkw1^* allele introduced from the C3HeB/HeJ substrain (FeJ.HeJ-*Gria4^spkw1^*F_1_ hybrids) were crossed to B6J mice with the *Gria4* insertion null allele (B6J-*Gria4^tm1Dgen^*) to generate an N_2_ population comprising 47 females and 42 males. None of these mice had the wild type *Gria4* allele, although, due to the design, approximately half were compound heterozygotes and half *Gria4^tm1Dgen/tm1Dgen^*. The C3HeB/FeJ substrain does not naturally contain the spontaneous *Gria4* mutation of the HeJ strain, and therefore does not experience the frequent SWD of the C3H strain (Tokuda et al. 2009). For the remainder of the manuscript, we refer to the FeJ substrain mice by the more common abbreviation of the strain C3H. For CAPE, the three datasets were combined as described below to ultimately contain 82 females and 108 males.

Tail-tip excision genomic DNA was prepared from each backcross or intercross animal, and single nucleotide polymorphism (SNP) genotyping at a marker density of approximately 15 Mb was provided by Kbioscience (currently LGC Genomics, LLC. Beverly, MA), using a custom panel. The final set of markers used in CAPE analysis is provided in Supplemental Table 1.

### EEG

Adult mice aged between 6 and 9 weeks were anesthetized with tribromoethanol (400 mg/kg i.p.) Small burr holes were drilled (1 mm anterior to the bregma and 2 mm posterior to the bregma) on both sides of the skull 2 mm lateral to the midline. Four teflon-coated silver wires were soldered onto the pins of a microconnector (Mouser Electronics, Texas). The wires were placed between the dura and the brain and an inorganic-based dental cap was then applied. The mice were given a post-operative analgesic of carprofen (5 mg/kg subcutaneous) and allowed a minimum 48 h recovery period before recordings were made. The mice were connected to the EEG Stellate Lamont Pro-36 programmable amplifier (Lamont Medical Instruments, Madison, WI) using a flexible cable with free access to all corners of the cage, including food, for 2 hr periods on 2 consecutive days (4 hr total), during the middle of the light cycle (typically between 9 AM and 4 PM). EEG data were collected with the software program Stellate Harmonie (Stellate Systems, Inc., Montreal, Canada). SWD episodes were scored visually using the following criteria: the EEG recording showed a SWD discharge that lasted for at least 0.5 seconds, with amplitude at least 2-fold higher than background, and observed concurrently in the majority of the 6 recording channels.

### Combining Data from Multiple Genetic Crosses

SWD incidence (SWD/hr) and length (s) were measured on two different days, and the average of the two days was used in this analysis. Each phenotype was mean-centered and adjusted with Z-transform normalization within each cross separately, and the crosses were combined into one data set containing 190 individuals. The same 191 genetic markers were measured across the genome in each of the three crosses. We recoded the entire meta-cross as a backcross for two main reasons. First, two of the three contributing crosses (those containing the *Scn8a* and *Gria4* mutations), were backcrosses. Second, very few loci in the intercross were homozygous for C3H (Supplemental Figure 1). The backcross encoding we used assumed dominance of the C3H allele, i.e. homozygous B6J were coded as 0 and both heterozygous and homozygous C3H were coded as 1. By coding the combined cross in this manner, we increased power to detect dominant effects of C3H alleles; however, we lost the ability to detect additive and recessive effects. Given the small number of mice that were homozygous for C3H alleles, the gain in power to detect dominant and interaction effects outweighed the loss in power to detect additive effects.

### Combined Analysis of Pleiotropy and Epistasis

The CAPE approach was first described in a yeast study (Carter et al. 2012). An R implementation has more recently been released and applied to a mouse intercross study (Tyler et al. 2013). CAPE is a strategy that both detects and interprets epistasis in terms of directional influences between genetic loci. The algorithm first performs linear regression on each locus pair for each trait in the cross. It then combines the results of the linear regressions across phenotypes to interpret the direction of the interaction. This directional information is calculated through a reparametrization of the coefficients from the pairwise linear regressions. The result is a pair of directed coefficients describing how the two loci influence each other in terms of suppression or enhancement.

Prior to calculating the linear regression models, we decomposed the phenotypes via singular value decomposition (SVD) into their principal components, called eigentraits (ETs). This step de-correlates the phenotypes, reorganizing phenotypic signals into orthogonal, composite phenotypes. This procedure potentially concentrates genetic associations, in that variants with weak associations to multiple original phenotypes often exhibit strong association to one ET. Although the final CAPE-derived model will be recast in terms of the original phenotypes, this provides enhanced detection of candidate loci for interaction analysis.

We filtered marker pairs based on linkage. High Pearson correlation between markers indicates that the individual markers supply redundant information and can lead to false-positive interactions. We included only those marker pairs with a Pearson correlation r ≤ 0.75.

We then performed a pairwise linear regression on all pairs of filtered markers 1 and 2:

$$U_{i}^{j}=\beta_{0}^{j}+\underset{\text{covariate}}{\underset{︸}{{\text{sex}}_{i}\beta_{\text{sex}}^{j}}}+\underset{\text{main}\;\text{effects}}{\underset{︸}{x_{1,i}\beta_{1}^{j}+x_{2,i}\beta_{2}^{j}}}+\underset{\text{interaction}}{\underset{︸}{x_{1,i}x_{2,i}\beta_{12}^{j}}}+\varepsilon_{i}^{j},$$

where *U* represents ETs, and *ε* is an error term. The index *i* runs from 1 to the number of individuals, and *j* runs from 1 to 2 (the number of ETs.) *x_i_* is the probability of the presence of the C3H allele for individual *i* at locus *j*. For all regression models, the coefficient of determination was computed as $R^{2}=\frac{{SS}_{\mathrm{reg}}}{{SS}_{\mathrm{tot}}}$. Where *SS_reg_* is the sum of squares of the regression and *SS_tot_* is the sum of squares of the dependent variable.

The coefficients from the pairwise regression are then reparametrized to give direct influence coefficients, describing how each marker either enhances or suppresses the activity of each other marker. First two new parameters (*δ*_1_ and *δ*_2_) are defined in terms of the interaction coeffici1`-ents from the pairwise regression. *δ*_1_ can be thought of as the additional genetic activity of the variant near marker 1 when the variant near marker 2 is present. The *δ* terms are independent of phenotype and together completely describe the interaction term. They can be interpreted as the extent to which each marker influences the effect of the other on the phenotypes. For example, a negative *δ*_2_ indicates that the presence of variant 2 suppresses the effect of variant 1 on the ETs. The *δ* terms are related to the main effects and interaction effects as follows:

$$[\begin{array}{c} \delta_{1}\\ \delta_{2} \end{array}]={\left[\begin{array}{cc} \beta_{1}^{1} & \beta_{2}^{1}\\ \beta_{1}^{2} & \beta_{2}^{2} \end{array}\right]}^{-1}\cdot \left[\begin{array}{c} \beta_{12}^{1}\\ \beta_{12}^{2} \end{array}\right]$$

The *δ* terms are then translated into directed variables defining the marker-to-marker influences *m*_12_ and *m*_21_. The term *m*_12_ is the direct influence of marker 2 on marker 1, where a negative value indicates suppression and a positive value indicates enhancement. The terms *m*_12_ and *m*_21_ are defined in terms of *δ*_1_ and *δ*_2_:

$$\delta_{1}=m_{12}(1+\delta_{2}),\;\delta_{2}=m_{21}(1+\delta_{1})$$

The significance of these influences is determined through standard error analysis on the regression parameters (Bevington 1994; Carter et al. 2012). Since matrix inversion can lead to large values with larger standard errors, this step is particularly important in determining which of these values are significant. We propagate the errors using a second-order Taylor expansion (Bevington 1994).

To calculate p-values for the directed influence coefficients we performed permutation testing. For each permutation, the two markers being tested in the regression were permuted together. This process retains any linkage between the two markers, but randomizes them relative to all phenotypes and covariates. Permutations were combined across all pairs to generate a single null distribution. This distribution is indistinguishable from the null distribution created by permuting any given pair many times, and combining across pairs reduces the number of total permutations that need to be performed. For the combined cross, we performed 33 permutations on each of 15,110 locus pairs yielding a total of 498,630 permutations.

The significance of the main effect of each locus was determined by the same permutations, with standardized main effects compared to a null distribution composed of the corresponding parameters in the permuted data. Each locus was tested for a main effect each time it was paired with another locus, giving each locus a distribution of main effects in these multiple contexts. To summarize each marker’s effect as a single number, the maximum effect of each locus was selected, and all p-values were corrected for multiple testing using a false discovery rate (FDR) of 0.05. Finally, all variant-to-eigentrait effects were multiplied by the singular value matrices from the original SVD to yield main effects and standard error estimates of the variants on the original phenotypes.

### Grouping Linked Markers

To define distinct QTL regions in the interaction network, adjacent markers were combined into linkage blocks. The first marker on a chromosome was assigned to the first linkage block. We then stepped along the chromosome comparing each subsequent marker to the first marker until we found one with a Pearson correlation *r* ≤ 0.5. This marker then became the first marker in the next linkage block, and the process was repeated until all makers were assigned to a linkage block. The value 0.5 was chosen to give a conservative estimate of linkage blocks. A block was considered to have a significant effect on a phenotype if one or more of the resident markers had a significant effect. After this point, we refer to linkage blocks with significant associations as QTL or loci. The main effect traces of all markers as determined by CAPE can be seen with the linkage block delineations in Supplemental Figure 2. Approximate locations of each block are listed in Table 2.

## Results

### Effect of C3H Strain Background on SWD Severity of Three Epilepsy Mutations

**Figure 1:**
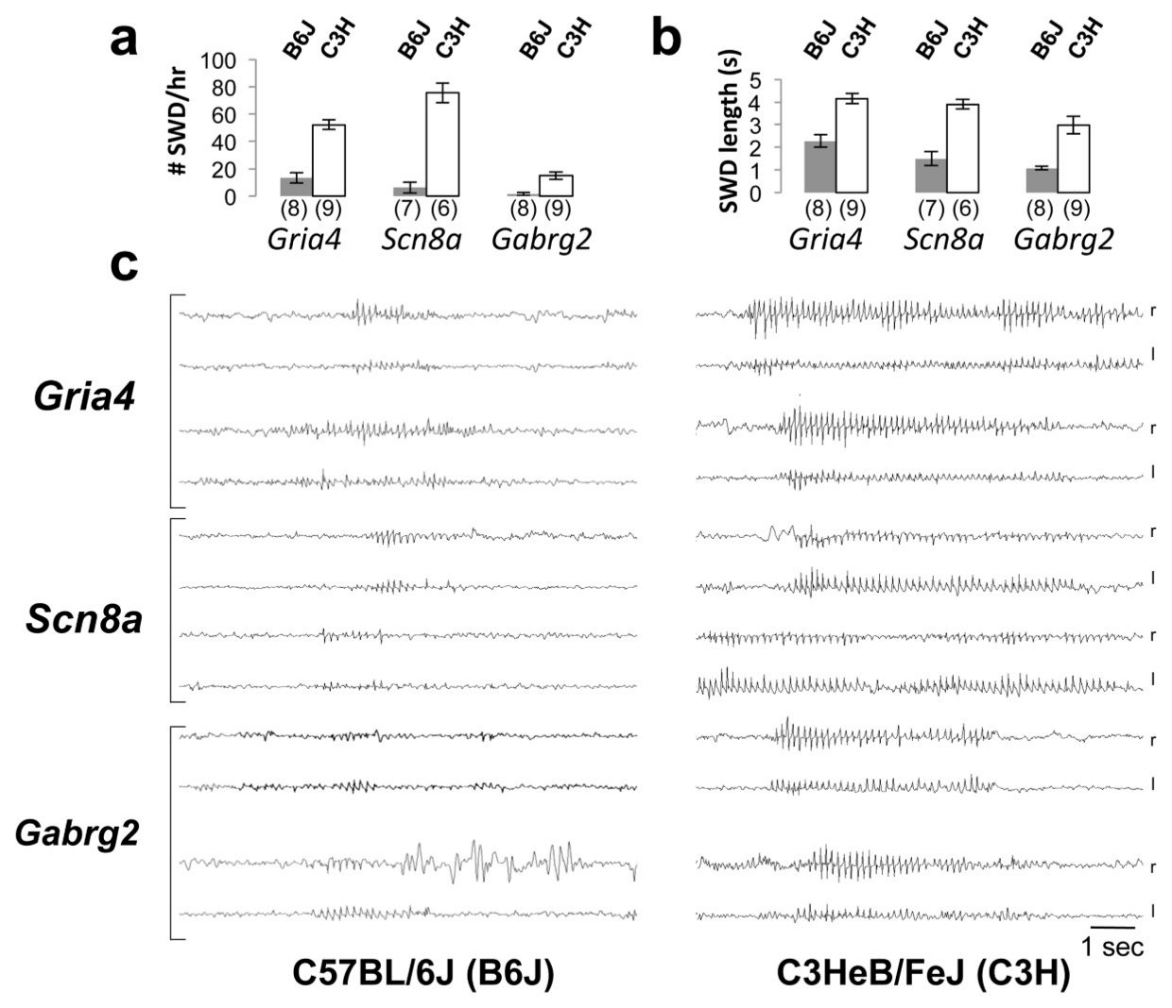
SWD incidence, length and morphology for each mutation in the B6J and C3H backgrounds SWD incidence (a) or mean SWD length (b) for each of three absence epilepsy mutant genotypes - *Gria4*, *Scn8a* or *Gabrg2* (Methods) - on the C57BL/6J (B6J) compared with C3HeB/FeJ (FeJ) mouse strain backgrounds. Error bars are SEM. Number of animals for each are shown in parentheses. The *Scn8a* and *Gria4* results are replotted data from related manuscripts (Oliva et al. 2014; Frankel et al. 2014, respectively); the *Gria4* and *Gabrg2* results are from new data in the present study. (c) Comparison of morphology for two representative SWDs from each mutation and strain combination, as indicated. For each, two of the six EEG channels are shown: “r” is right hemisphere, “l” is left hemisphere – each a differential signal from an electrode placed about 1 mm rostral to one placed about 1 mm caudal, relative to the Bregma suture.

**Figure 2:**
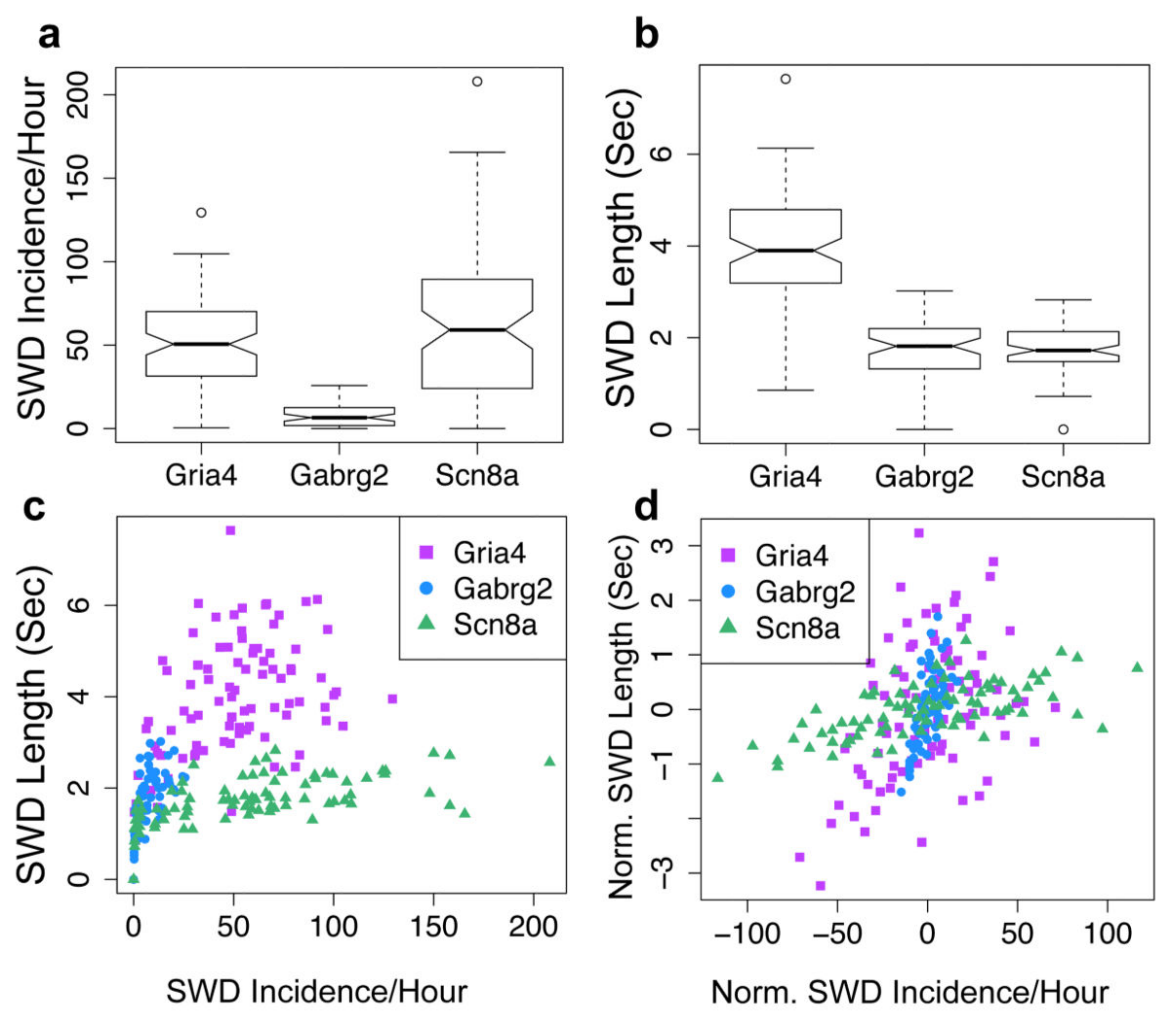
Distributions of SWD incidence and length in the three crosses each labeled by mutated gene (a) The distribution of raw SWD incidence (SWD/hr) in each cross. (b) The distribution of raw SWD length (seconds) in each cross. (c) The correlation between raw SWD incidence and length. D) The correlation between SWD incidence and length after the normalization and mean-centering procedure. The Pearson correlation coefficient (r) between the normalized SWD incidence and length is 0.44 for data combined across all crosses.

To confirm and extend the observations that the C3H mouse strain background facilitates more severe and frequent SWD in multiple absence seizure-associated mutations, we backcrossed each of three mutations, to the C3HeB/FeJ (C3H) and to the C57BL/6J (B6J) inbred strains and examined SWD incidence and average length from EEG recordings for each respective congenic strain. As expected from previous studies with partially backcrossed mice, SWD measures were significantly enhanced after the *Gria4* and *Scn8a* mutations were fully backcrossed to the C3H strain compared with B6J (Figure 2a,b). Interestingly, we found that *Gabrg2^R43Q^* also showed a similar effect in the same direction (Fig. 1a,b). The SWD morphologies in each also tended to be strongest on the C3H strain; there were more regular spike-wave complexes with typically high amplitudes when compared with the same mutation on B6J (Figure 1c).

### Segregation of SWD measures in C3H x B6J second generation progeny: Distribution and Correlation

Individuals from backcross and intercross populations were subjected to EEG analysis and genome scan as described in the Methods. The phenotype measures, SWD length and SWD incidence, varied between three cross populations; for example, the average SWD incidence in the *Gabrg2* cross was low relative to the other two crosses (Figure 2a), and the *Gria4* cross had a relatively high average SWD length with high variance (Figure 2b). To put the crosses into a comparable field, we normalized and mean-centered the crosses as described in Methods, thus generating a “meta-cross” for further analysis (Figure 2c,d).

### Single-Locus Effects on SWD Incidence and SWD Length

We performed a single-locus interval mapping analysis in the meta-cross using imputed pseudomarkers derived from the hidden Markov process provided in R/qtl (Broman et al. 2003). This single-locus analysis of direct SWD measures shows that a region on Chr 2 is significantly associated (*p* < 0.05) with SWD incidence (Supplemental Figure 3). The region also has a peak for SWD length, but the effect does not reach significance. The association between Chr 2 and SWD incidence was the only significant association. We also performed single-locus QTL analysis in each of the individual crosses using R/qtl (Broman et al. 2003). In the cross containing *Gria4* mutant genotypes, there was a single significant QTL on Chr 15 (Frankel et al. 2014). A similar analysis of the cross containing the *Scn8a* mutation detected Chr 7 as a main effect (Oliva et al. 2014).

### Single-Locus Effects on Eigentraits

We used singular value decomposition (SVD) to decompose the two SWD measures in the meta-cross into two orthogonal eigentraits (ETs). The first ET represented the common features between SWD length and incidence while the second ET represented features that are disparate between the two phenotypes. Both ETs were used in the analysis.

**Figure 3:**
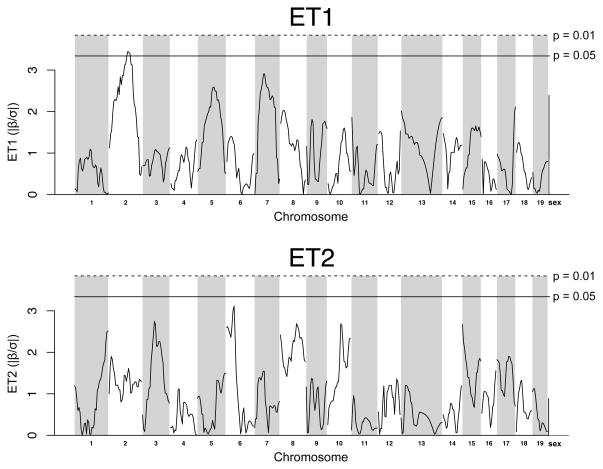
Single-locus scans of ET1 and ET2 for meta-cross The standardized effects (β/σ) of each locus is shown for each eigentrait (ET). ET1 is the sum of the normalized SWD incidence and length phenotypes, whereas ET2 is the difference. Significance levels were determined by 100 permutations of the data. For this single-locus scan, pseudomarkers were imputed between the genotypes markers using R/qtl (Broman et al. 2003).

We then performed a genome-wide, single-locus regression to determine the extent to which each locus influences each of the ETs. QTL associated with these ETs are pleiotropic influencing both SWD incidence and length. QTL associated with the first ET represent positively pleiotropic loci, where the C3H alleles have the same influence on both SWD incidence and length, i.e. the locus affects both either positively or negatively. Only one significant association was detected. A region on Chr 2 was significantly associated with ET1 (*p* < 0.05) indicating it influences both SWD incidence and length (Figure 3). This QTL is similar to that observed for the SWD measures in the meta-cross discussed previously (e.g. Supplemental Figure 3).

Some of the markers used were tightly linked to one another. High Pearson correlation between markers indicates that the individual markers supply redundant information and can lead to false-positive interactions. To address this problem, we filtered the marker pairs by removing all highly correlated pairs from consideration using a Pearson correlation cutoff of *r* ≥ 0.75. This threshold removes the most highly correlated pairs, but is liberal enough to include markers that may interact despite a moderate correlation. A total of were 466 marker pairs were eliminated in this filtering step leaving a total of 15,110 marker pairs to be tested in the pairwise scan.

### A Genetic Interaction Network Influencing SWD Incidence and Length

**Figure 4:**
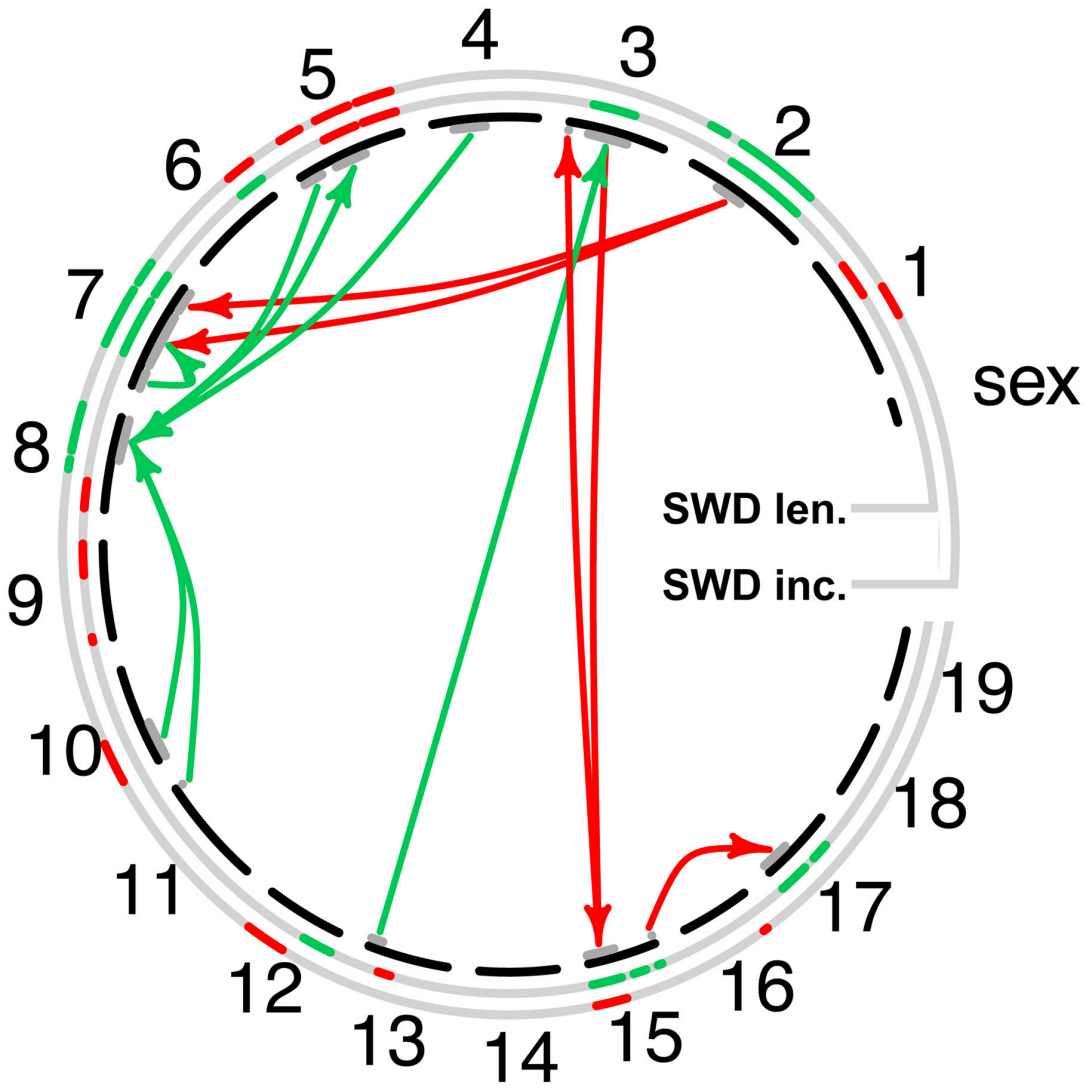
Network of Genetic Interactions Between QTL Each chromosome is represented by a black bar and labeled with the chromosome number. Main effects are depicted in the gray circles outside the chromosome bars. Green segments represent significant positive main effects and red segments represent significant negative main effects. The inner and outer main effect circles show main effects for SWD length and SWD incidence respectively. Interactions are represented by arrows between chromosomal segments. The chromosomal segment involved in the interaction is shown as a gray bar on the inside of the black chromosomal bar. Four sub-networks of individual modules can be seen as arrows linking common regions together. One subnetwork links Chr 8 to Chrs 4, 5, 10 and 11. Another links Chrs 3, 13, and 15. Two smaller subnetworks link Chrs 2 and 7 into one module and Chrs 15 and 17 into another module. All effects are for C3H genotypes at each QTL.

After filtering the marker pairs, we used CAPE to determine the contribution of genetic interactions to the variation in phenotypes. To avoid the burden of multiple correlated tests, we used only genotyped markers and discarded the pseudomarkers used for single-locus interval mapping. We detected multiple genetic interactions of genome-wide significance that had not been detected with the pairwise regression-based method. The interactions form a directed network comprising 10 chromosomes (Figure 4). Main effects in this network are indicated by colored bars outside the black chromosomal bars, and interactions are denoted by arrows between chromosomal regions. All interactions for individual markers are listed in Supplemental Table 3.

In the meta-cross there was a mixture of suppressive and enhancing interactions. These interactions were distributed across four distinct modules that can be seen here as interactions sharing individual chromosomal regions (Figure 4). The largest module, for example comprises positive interactions between Chrs 4, 5, 8, 10, and 11 with Chr 8 acting as the hub of the module. The module including Chrs 2 and 7, as well as the module connecting Chrs 15 and 17 both linked chromosomal regions with positive main effects. Two other modules, one linking Chrs 3, 13, and 15, and the other linking Chrs 4, 5, 8, 10, and 11, however include mixtures of positive and negative main effects on the two phenotypes.

The modules also displayed different mixtures of positive and negative interactions. Two multi-node modules contained both enhancing and suppressing interactions, while one other module contained only positive interactions. The latter module has a clear hub on Chr 8. This chromosome had a positive effect on SWD incidence, which was enhanced through interactions with loci on four other chromosomes.

**Figure 5:**
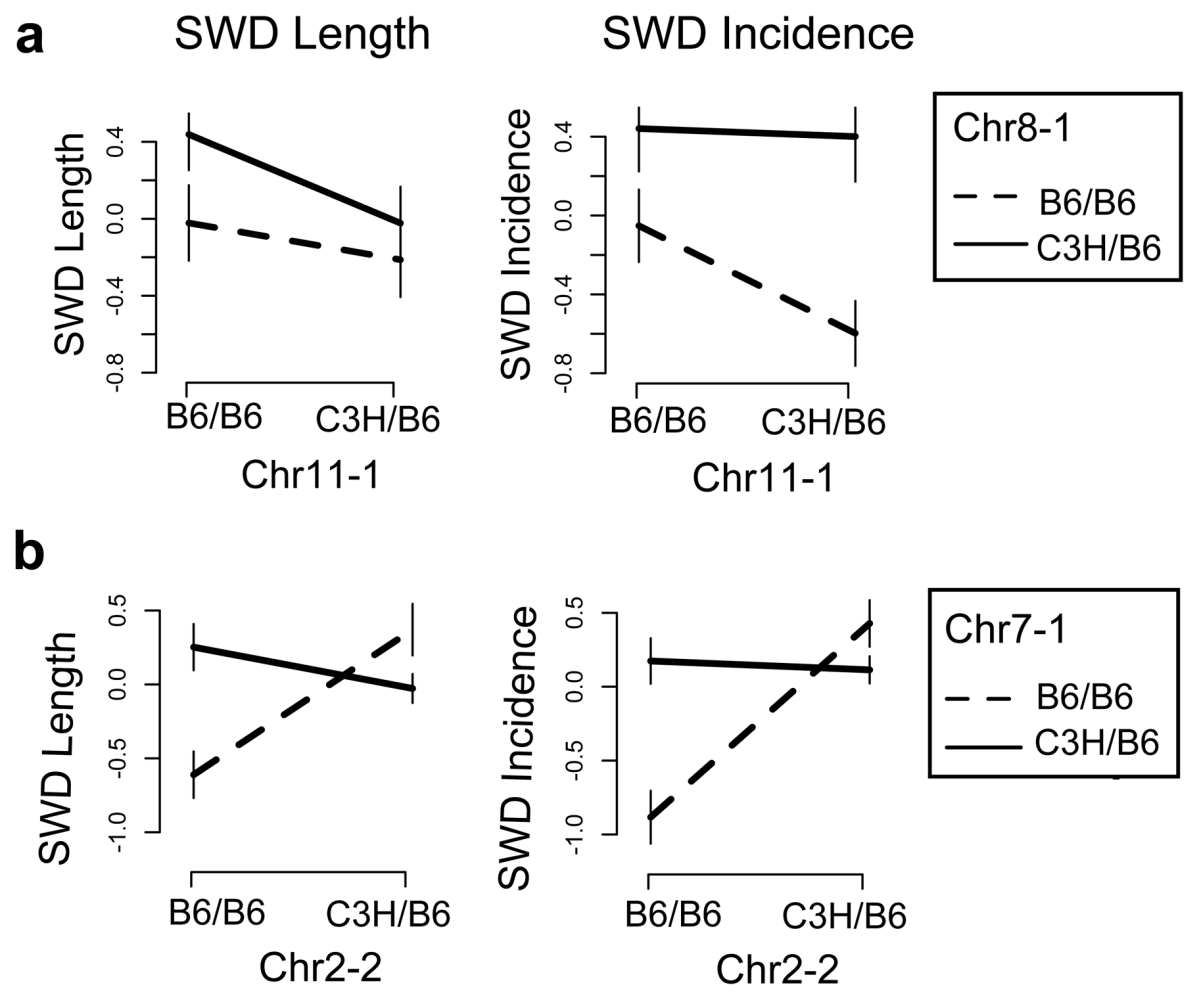
Examples of Enhancing and Suppressing Interactions (a) Effect plots for an enhancing interaction between the first linkage block on Chr 8 and the first linkage block on Chr 11. The Chr 11-1 locus has no independent main effects, but it enhances the positive effect of the Chr 8-1 locus on SWD incidence. When the Chr 11-1 locus has the B6J genotype, the Chr 8-1 locus has a small positive effect on SWD incidence. However, when the Chr 11-1 locus has the C3H genotype, the Chr 8-1 locus has a large positive effect on the phenotype. (b) Effect plots for a suppressing interaction between the second linkage block on Chr 2 and the first linkage block on Chr 7. Both regions have positive main effects on both phenotypes. However, the Chr 7-1 locus only has a positive influence on the phenotypes when the Chr 2-2 locus has the B6J genotype. If the Chr 2-2 locus has the C3H genotype, it completely suppresses the positive effect of the Chr 7-1 locus, and even reverses the effect to be slightly negative.

The nature of this enhancement can be seen by examining effect plots for these interactions. For example, in the interaction between the first chromosomal segment on Chr 11, and the first on Chr 8, the C3H allele on Chr 8-1 has a small positive effect in animals with the B6J background on Chr 11-1. But in animals with the C3H allele on Chr 11-1, the positive effect of the Chr 8-1 allele is much larger (Figure 5a). The maximum SWD incidence is not increased in animals with both C3H alleles, but rather there is a relative increase in the effect of the Chr 8-1 C3H allele across Chr 11-1 genotypes. The result is an enhancing interaction even though the SWD incidence in animals with the C3H allele on Chr 8-1 is constant across Chr 11-1 genotypes.

Suppressive interactions are also easily interpreted through effect plots. For example, in one module, Chr 2 suppressed the effects of Chr 7 (Figure 5c). The effect plot shows that the C3H allele on Chr 7 has a large positive effect in animals with the B6J genotype on Chr 2. However, this positive effect is completely suppressed, and even slightly reversed in animals with the C3H allele on Chr 2. All interaction plots can be seen in Supplemental Figures 4a and b.

We estimated the phenotypic variance explained by each model to assess the impact of the interactions. These calculations can be directly computed from the regression fits, which are unchanged by our model reparametrization. The interacting models had high coefficients of determination (median *R*^2^ = 0.083 for SWD length and median *R*^2^ = 0.12 for SWD incidence), compared to median values from the corresponding non-interacting pair-wise models (*R*^2^ = 0.036 for SWD length and *R*^2^ = 0.088 for SWD incidence). Although the interacting models invariably fit more variance, an analogous comparison of non-significant models yields only modest improvement in fit for the interacting model (median *R*^2^ = 0.022 versus *R*^2^ = 0.015 for SWD length; median *R*^2^ = 0.022 versus *R*^2^ = 0.012 for SWD incidence).

### Distinguishing Pleiotropy from Conditional Pleiotropy

Although the single-locus scan can identify potentially pleiotropic loci, CAPE can distinguish between QTL that are independently pleiotropic and QTL that participate in conditional pleiotropy. We define a conditionally pleiotropic locus as one that independently influences only phenotype A, but conditionally influences phenotype B by interacting with a second locus that affects phenotype B. The first locus does not have an independent effect on phenotype B, but it changes the effect of the second locus on phenotype B, thereby indirectly influencing this phenotype.

**Figure 6:**
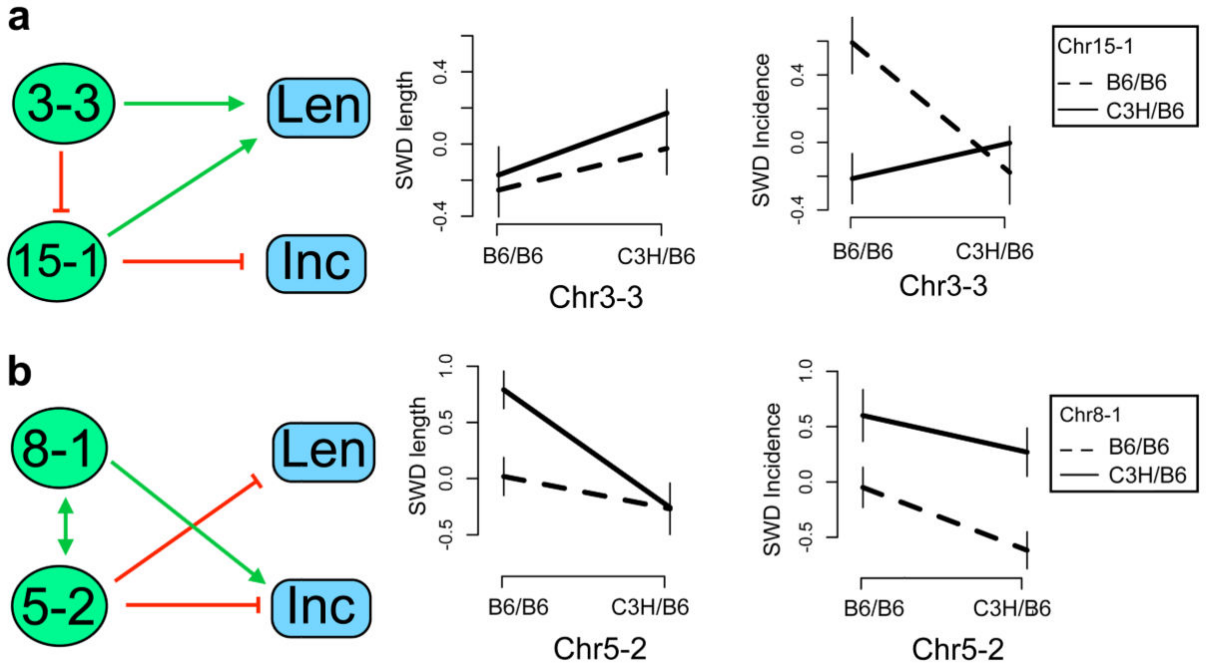
Examples of Conditional and Independent Pleiotropy (a) An example of conditional pleiotropy. Chr 3 block 3 has an independent positive main effect on SWD length and suppresses a QTL on Chr 15 block 1, which has a negative main effect on SWD incidence. Effect plots (right panel) illustrate how the Chr 3-3 QTL has a conditional influence on SWD incidence by suppressing the negative effect of the QTL on Chr 15-1. When the genotype on Chr 3-3 is B6J, Chr 15-1 has a large negative effect on SWD length. However, when the C3H allele is present on Chr 3-3, the genotype at Chr 15-1 has no effect on SWD length. In this way, the Chr 3-3 genotype has an indirect effect on SWD length. (b) An example of independent pleiotropy. The QTL on Chr 5 block 2 has an independent effect on both SWD length and incidence. This main effect is evident in the effect plots (right panel) as the heterozygote has a lower value of both phenotypes than the B6J homozygote.

Chr 3 is a good example of conditional pleiotropy. It has a clear independent positive effect on SWD length and no overall effect on SWD incidence (Figure 6a). However, Chr3 does have a conditional effect on SWD incidence when its influence is considered jointly with that of Chr 15 (Figure 6a). Chr 3 has a strong negative effect on SWD incidence only when the genotype on Chr 15 is B6J. When the background genotype on Chr 15 is C3H, the genotype on Chr 3 has no effect on SWD incidence. On the other hand, the Chr5 QTL interacting with Chr 8 has very clear independent negative effects on both phenotypes (Figure 6).

## Discussion

The genetic complexity of absence epilepsy (AE) has been an obstacle in elucidating its molecular basis. A few causal genes have been identified in both humans and rodent models, but evidence suggests that further progress requires direct assessment of genetic interactions modifying AE phenotypes. Here we examine genetic interactions across three mouse models of AE using CAPE, a novel approach for detecting and interpreting epistasis. With this approach we demonstrated increased power to detect genetic interactions and main effects compared with regression-based techniques. Furthermore, the combination of molecular mechanisms in the population, provided the opportunity to identify broadly relevant genetic modifiers of AE phenotypes.

In this analysis, CAPE had a clear advantage in power over standard regression-based strategies. The latter detected only one significant main effect of Chr 2 for one of the two SWD phenotypes – SWD incidence – and no significant pairwise interactions at p ≤ 0.05. However, at the same significance level CAPE revealed multiple significant loci with main effects and a complex network of directed genetic interactions between loci influencing both SWD incidence and length. Comparing the two methods in the individual crosses showed that interval mapping detects only the strongest effects found by CAPE. For example, interval mapping in the cross containing the *Gria4* mutation, found a significant main effect on Chr 15 (Frankel et al. 2014). Although interval mapping did not detect this region in the meta-cross, CAPE did detect it. A similar interval mapping analysis of the cross containing only the *Scn8a* mutation separately detected a main-effect QTL on Chr 7, which was shown to be an interacting region by CAPE in the meta-cross (Oliva et al. 2014). Both the Chr 15 (Frankel et al. 2014) locus and Chr 7 locus (Oliva et al. 2014) were validated using a congenic strain in which the C3H-derived susceptible region was placed into a B6J strain background. Although these findings are helpful in identifying associated regions, these conventional methods are often difficult to interpret when QTL are inconsistent due to underpowered studies or genetic background effects. CAPE improves both of these limitations by (i) increasing power through integrating information in both phenotypes, and (ii) accounting for genetic interactions.

The meta-cross used in this study is advantageous in that it increases the size of the population and improves statistical power in our study. Although meta-populations are often underpowered compared to studies of single populations of comparable size, our study circumvented some of the usual drawbacks of meta-populations. Frequently, meta-populations are constructed from sub-populations of human subjects, each of which has distinct genetic substructure. This can lead to false negatives for associations specific to one sub-population and false positives due to genetic similarity within each sub-population. In this study, all mice were derived from the same parental strains, thereby eliminating this limitation. Furthermore, all mice were phenotyped in a single laboratory, eliminating a common source of variation. To address remaining batch effects, we mean centered and normalized within each sub-population. This procedure reduces the incidence of false positive associations, although it might also reduce power to detect true positives of exceptionally large effect. An additional limitation of our meta-cross was that engineered mutations were restricted to individual sub-populations and therefore did not segregate, precluding the inference of interactions between these mutations.

Other potential complications in the data come from genetic segments linked the mutations of interest in each sub-population, stemming from two sources. First, two of the mutations (*Gabrg2*^tm1Spet^, *Gria4*^tm1Dgen^) were constructed in the 129 strain before backcrossing began, potentially substituting B6 alleles with 129, which might be functionally shared with C3H. Backcrossing to congenicity does not completely isolate the mutation of interest, and a segment of donor strain genetic material – so called “passenger” - usually remains after backcrossing is completed. However, in each subpopulation, backcrossing was performed for at least 10 generations. Theoretical work suggests that after 10 generations of backcrossing, the introgressed region is likely less than 10 cM (Hospital 2001), so although contamination is likely, the regions are unlikely to be large.

In our data, passenger material from 129 is likely to exist in the *Gria4* mutants, since the *Gria4* knockout was constructed in this background. The nearest marker (10 Mb away) does not distinguish between B6 and 129, so we cannot determine the passenger region within 10 Mb. However, the backcross for this mutation was performed for 10 generations to minimize the size of the introgressed region as much as practically possible. In the *Gabrg2* mutants, the nearest marker to the mutation (5 Mb away) was B6 for the population, indicating that the introgressed region was small. Finally, the *Scn8a* mutation was originally in the B6 background and should not contain a passenger segment. Furthermore, this region was not included in the interaction network and is thus of minimal concern. So although it is possible that passenger genes near the mutations of interest modify the phenotypes studied here, reasonable practical measures were taken to minimize passenger regions surrounding the mutations. Moreover, our goal is to prioritize candidate regions for further research and mapping, rather than to identify genes, so we assert that possible contamination is of minimal concern.

Second, congenic passenger segments aside, epistatic modifiers that happen to be closely linked to a sensitizing mutation, even when detected in other crosses, are impossible to detect in the ‘cis’ cross, because of transmission bias (e.g. *Scn8a* and *Gabrg2* mutant mice are by definition all heterozygous for closely linked genes). Detecting closely linked modifiers is an inherent problem in any experimental cross. However, from genomic sequence from B6, 129 and C3H strains, the mutant genes themselves do not appear to harbor nonsynonymous coding, splice site or structural variant polymorphisms.

The interactions detected by CAPE were both suppressive and enhancing. Suppressive interactions may represent redundancy in the genetic network. The enhancing interactions usually enhanced positive effects on phenotype and tended to take a particular form. Rather than increasing the phenotype overall, in enhancing interactions a C3H allele at one QTL tended to increase a moderate phenotype in the B6J background and a low phenotype in the C3H background to the same high level. This indicates that the C3H allele at the first locus generates an absolute phenotype and may act downstream of QTL at the second locus.

While the majority of the detected loci influenced either SWD incidence or length, multiple loci were pleiotropic, i.e. affected both SWD measures. Of these loci, most were positively pleiotropic, affecting both phenotypes in the same direction. Positive pleiotropy has been previously demonstrated as the predominant form of pleiotropy in studies of related traits (Leamy et al. 1999; Leamy et al. 2002; Cheverud 2000; Wolf et al. 2005). It may indicate common molecular pathways underpinning both aspects of SWD.

There were two loci, on Chrs 6 and 15, that exhibited negative pleiotropy. Both chromosomes had positive effects on SWD length, but negative effects on SWD incidence. Such loci may be important in understanding subtle aspects of SWD generation since they are suggested here to increase atypical SWD that are infrequent but long in duration.

Our analysis also revealed cases of conditional pleiotropy, in which a locus itself independently influences a single trait, but through its interaction with a second locus, is associated with additional traits when a variant is present at the second locus. In this network, CAPE distinguishes conditional pleiotropy from independent pleiotropy, improving the genetic model and suggesting underlying pathway structure. For example, in the interaction between the third linkage block on Chr 3 and the first block on Chr 15, we see that Chr 3-3 only influences SWD incidence through an interaction with Chr 15-1 suggesting that Chr 3-3 may act upstream of Chr 15-1 to influence SWD incidence. However, Chr 3-3 has an independent effect on SWD length, indicating that it may also participate in a separate pathway independent of the locus on Chr 15-1 to affect SWD length independently. These instances of conditional pleiotropy often appear as “epistatic pleiotropy” (Wolf et al. 2005; Wolf et al. 2006), in which pairs of QTL exhibit epistasis across multiple related traits. CAPE integrates these patterns of epistatic pleiotropy across traits to obtain a summary model of genetic etiology.

The significant interactions accounted for a substantial increase in the phenotypic variance explained. For SWD length, the generally lower *R*^2^ values along with the relatively greater increase due to interactions (Results) suggests that this phenotype is more genetically complex than SWD incidence, such that candidate loci are more difficult to detect unless interactions are included in the model. Although it is tempting to estimate a total variance by summing all models, all interaction models were independently fit. Therefore each pair-wise model includes main effects from two loci (in addition to the interaction) that would be repeatedly included in the sum. Furthermore, a number of interactions may encode redundant information of pathway structure (Carter 2013), further overestimating the explained variance. Nevertheless, our analysis provides evidence of how a network modeling approach can increase the variance explained and, moreover, provide interpretable models of genetic interaction. The directional interactions in the network should add further biological insight, especially when some of the underlying genes are identified. That Chrs 2 and 7 interact in a single module and have similar phenotypic effects suggests that the causative genes within these regions may function in the same pathway. The unidirectional interaction between these chromosomes may further suggest the ordering of the underlying molecular pathway when allele effects (hypo- or hypermorphic) are known (Avery and Wasserman 1992). The instances of bidirectional interactions in the network suggests that the order of the interacting chromosomes cannot be discerned. It is possible that the causative genes in these regions encode proteins that interact in a complex. Alternatively, inferring the directionality of these interactions may require measurement of additional phenotypes (Carter 2013).

The complex genetic hypotheses generated by our analysis may also help map epilepsy-related genes to human disease. Four GWA studies in the genome.gov database link human chromosomal regions to epilepsy. They identify genes on multiple human chromosomes including the Scn8a relative *SCN1A*. Although the large chromosomal regions in our study preclude direct comparison with human associations, the results presented here can direct future fine-mapping mouse studies to find genes and pathways that interact with known causative genes, which may in turn help map interacting genes in human epilepsies.

Our study identified key genomic regions for further analysis, interprets the interactions between these regions to help prioritize future experiments, and generate hypotheses about pathway ordering of multiple chromosomal regions. However, the regions identified here are too large to speculate on candidate genes. Future studies using more mice and denser genetic sampling combined with CAPE may be able to further resolve the genetic network. Alternatively, the use of CAPE with heterogeneous stocks involving more than two strains, such as the Diversity Outbred mice (Svenson et al. 2012) or other stocks (Huang et al. 2009; Johannesson et al. 2009) generated with seizure-susceptible mice, offers a promising route to further dissect the genetic heterogeneity of complex diseases such as absence epilepsy.

## Supplementary Material

Supp FigureS1-S4 **Figure 1. Genotype Frequencies at Each SNP Genotype** (B6J/B6J, B6J/C3H, or C3H/C3H) frequencies in the combined meta-cross. The homozygous C3H genotype (purple) is rare compared to the homozygous B6J genotype (blue) and heterozygous B6J/C3H genotype (green). For this reason we coded the genotypes assuming C3H dominance with homozygous B6J coded as 0 and both heterozygous and homozygous C3H were coded as 1. In the cross containing the Gria4 mutation there are two null Gria4 alleles present near the most proximal marker on Chr 9. One is the Gria4spkw1 allele and the Gria4tm1Dgen allele. Because these alleles are both null alleles that exhibit the same phenotype, they were coded identically.
**Figure 2. Main Effects Determined by CAPE with Linkage Blocks Superimposed.** The main effects for each genotyped marker determined by CAPE. Gray and white polygons indicate different chromosomes. Vertical gray lines indicate boundaries of the linkage blocks. Horizontal lines mark p values adjusted for multiple tests.
**Figure 3. Effects of Individual Loci on SWD Incidence and Length.** The standardized effect size (β/σ) shows the direction of effect of each locus on SWD length and incidence. Chromosomes are indicated by alternating gray and white bars, and chromosome numbers are marked along the x-axis.
**Figure 4. Interaction Plots Representing All Interactions Between Chromosomal Segments** (a–b) Each panel shows a representative interaction between two chromosomal regions. All significant interactions are plotted. The left-hand section of each figure shows a cartoon depiction of the interaction. Green arrows between chromosomal regions indicate enhancing interactions, and red arrows indicate suppressive interactions. Green arrows to phenotypes indicate positive main effects, and red arrows indicate negative main effects. The right-hand section of each panel shows the interaction plot for the interaction depicted in the cartoon. The normalized phenotype of each genotype group is shown with error bars showing standard error.

Supp TableS1 Table 1. SNPs Used for Analysis A comma-separated text file listing all single nucleotide polymorphisms (SNPs) genotyped for this analysis.

Supp TableS2 Table 2. Approximate Locations of Linkage Blocks A comma-separated text file listing the approximate locations in Mb of each linkage block as defined in the methods. Each region is defined by the first and last markers in the block. Because we do not use interval mapping to identify the locations of the interactions, these regions should be taken only as marker groups and not necessarily regions containing the QTL.

Supp TableS3 Table 3. Directed Genetic Influences Between Markers A comma-separated text file listing all directed genetic influences obtained from CAPE. The individual SNPs listed here were compressed by linkage to generate the network in Figure 4.

Supp TableS4 Table 4. Genotype and Phenotype Data A comma-separated text file formatted for use in CAPE. This file contains the genotype and phenotype data for the three crosses. The phenotypes have been mean-centered and adjusted with Z-transform normalization within each cross separately. The genotypes have been coded assuming dominance of the C3H allele. The genotype A indicates homozygous B6, and H indicates either heterozygous or homozygous C3H.
