# Supplementary figures and images for: A Genetic Interaction Network Model of a Complex Neurological Disease
Source: Genes Brain Behav. Author manuscript; Available in PMC 2015 Nov 1. (PMC4241132; doi:10.1111/gbb.12178)

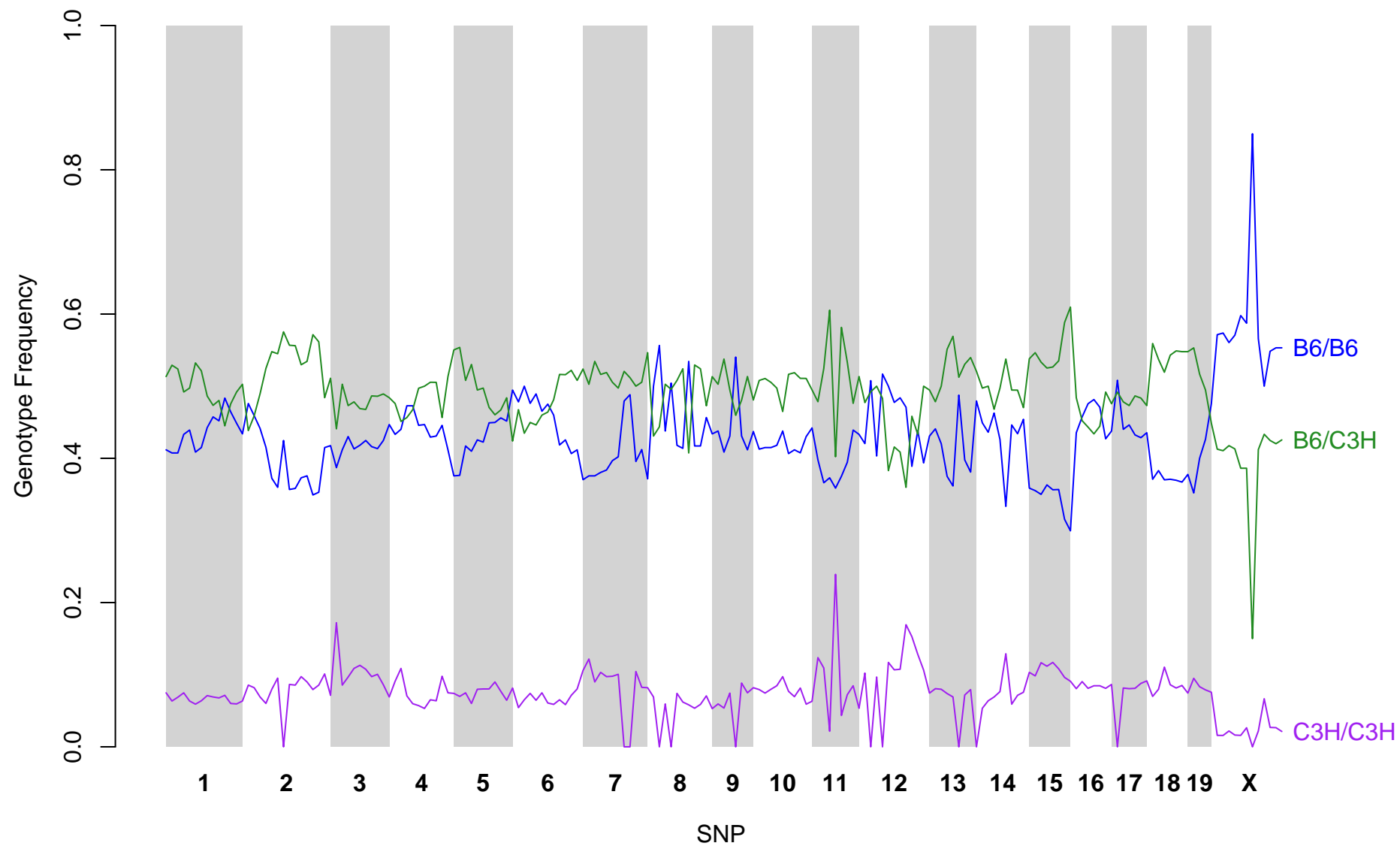

# SWD Length

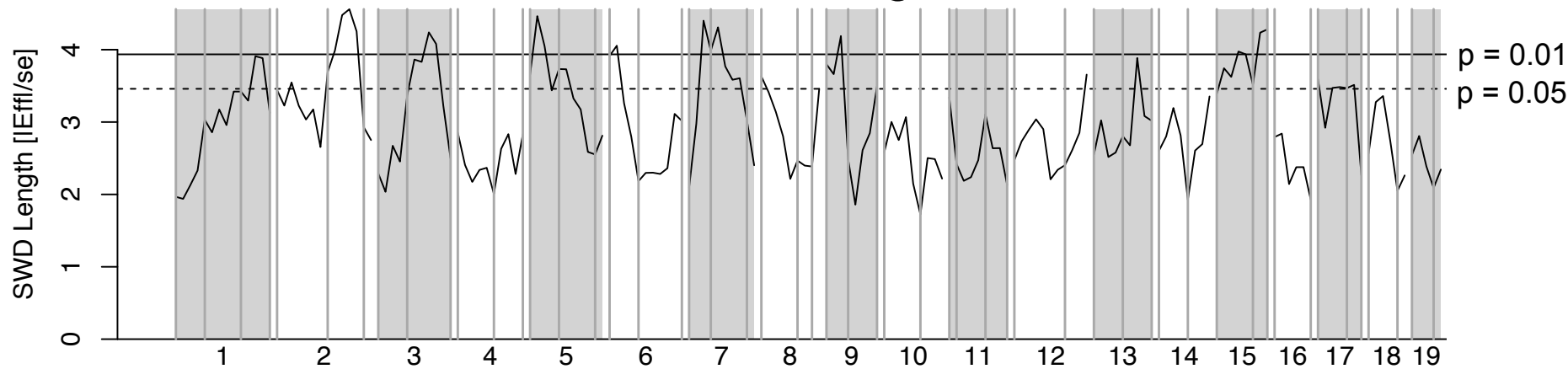

# SWD Incidence

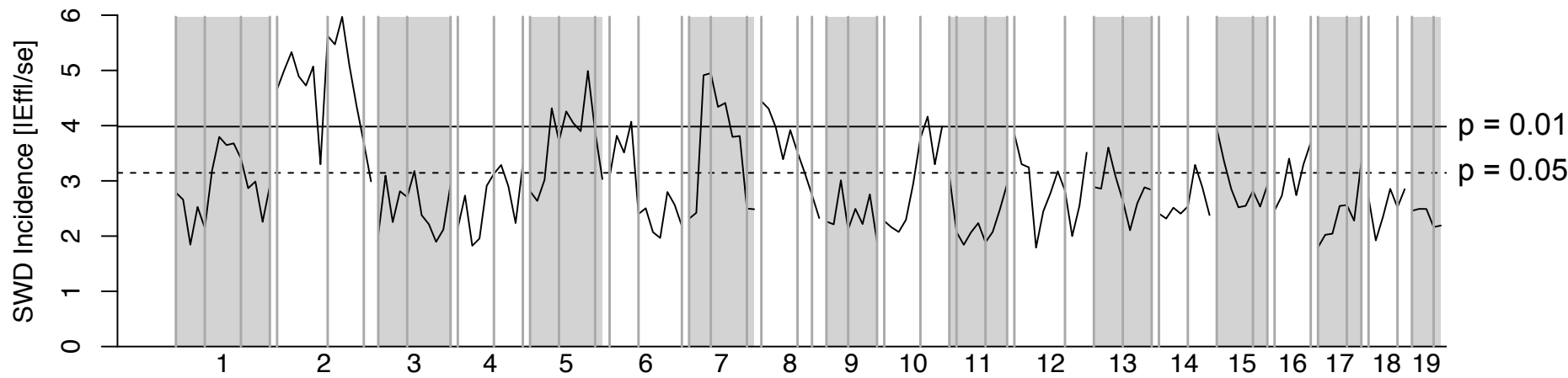

# SWD Length

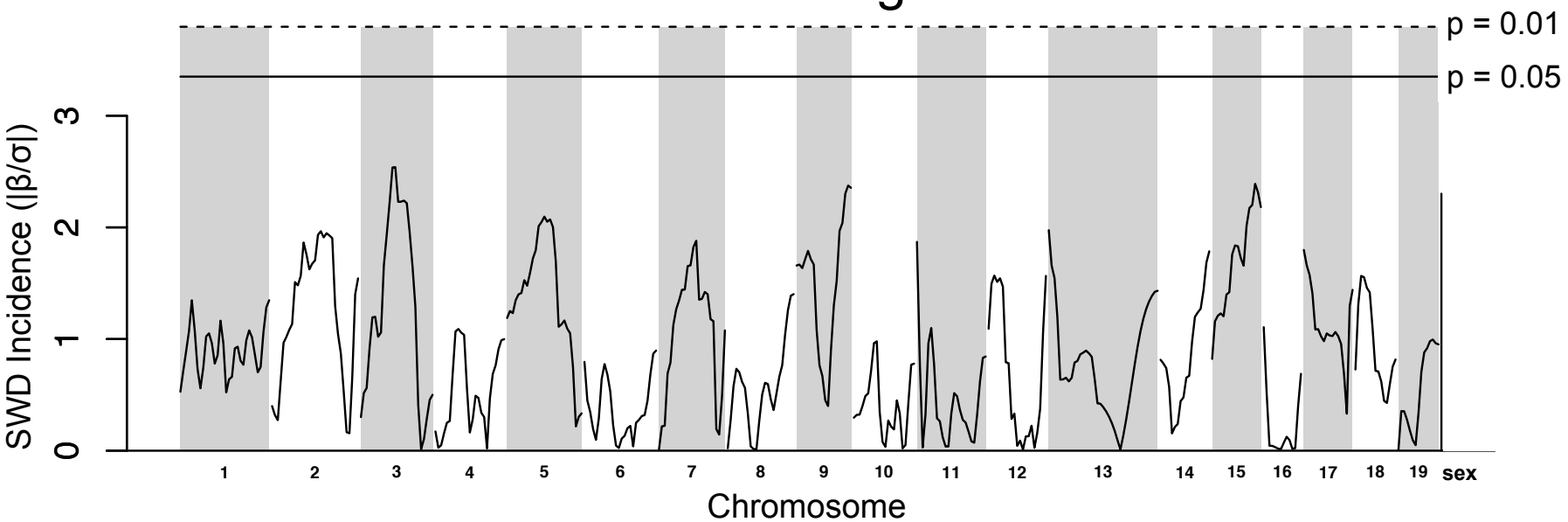

# SWD Incidence

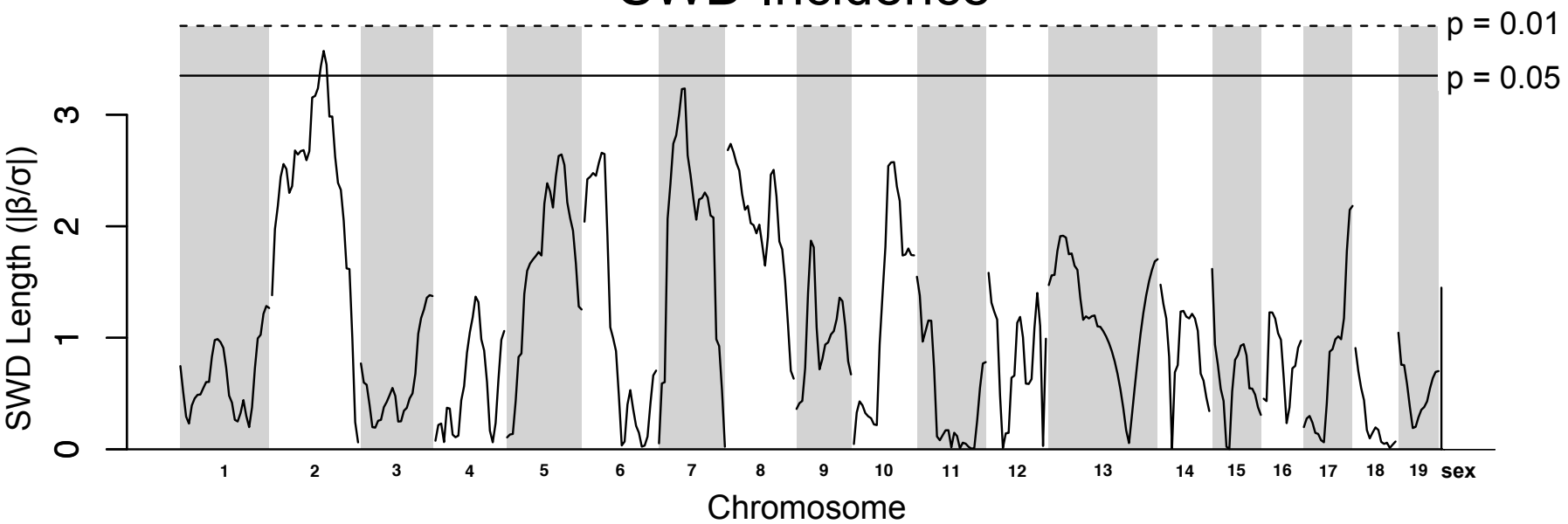

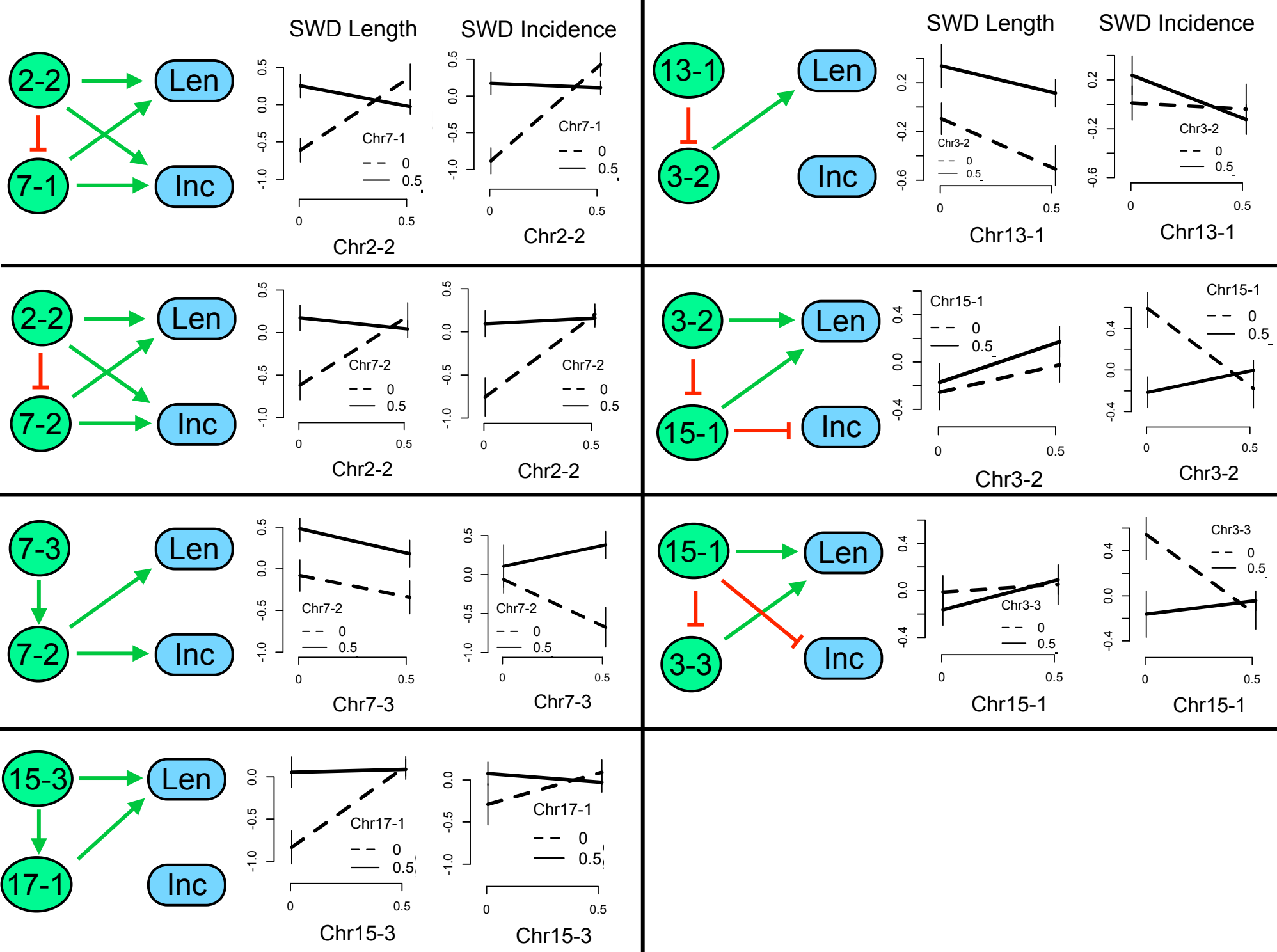

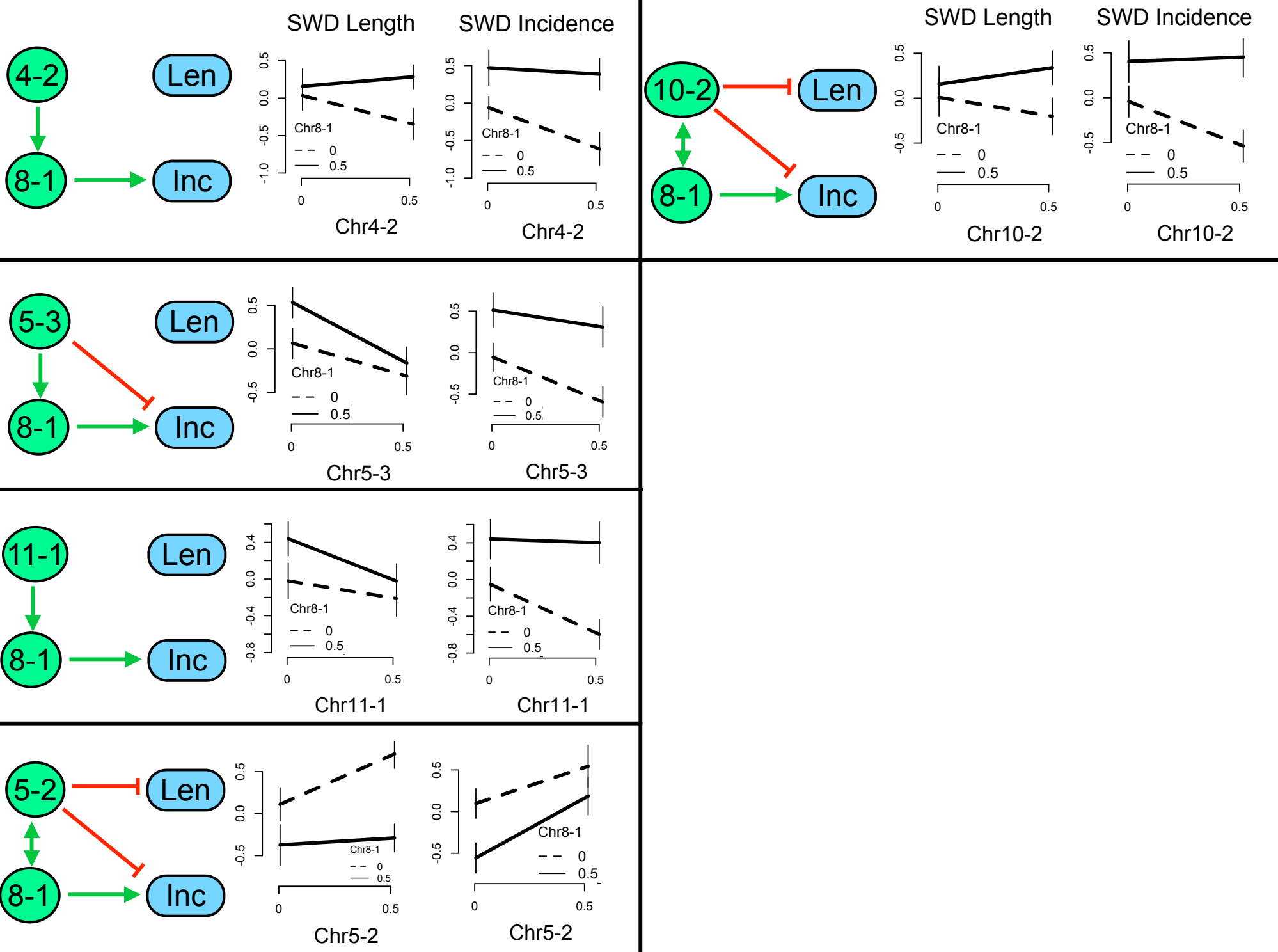

Supplement: Supp FigureS1-S4 — Figure 1. Genotype Frequencies at Each SNP Genotype (B6J/B6J, B6J/C3H, or C3H/C3H) frequencies in the combined meta-cross. The homozygous C3H genotype (purple) is rare compared to the homozygous B6J genotype (blue) and heterozygous B6J/C3H genotype (green). For this reason we coded the genotypes assuming C3H dominance with homozygous B6J coded as 0 and both heterozygous and homozygous C3H were coded as 1. In the cross containing the Gria4 mutation there are two null Gria4 alleles present near the most proximal marker on Chr 9. One is the Gria4spkw1 allele and the Gria4tm1Dgen allele. Because these alleles are both null alleles that exhibit the same phenotype, they were coded identically. Figure 2. Main Effects Determined by CAPE with Linkage Blocks Superimposed. The main effects for each genotyped marker determined by CAPE. Gray and white polygons indicate different chromosomes. Vertical gray lines indicate boundaries of the linkage blocks. Horizontal lines mark p values adjusted for multiple tests. Figure 3. Effects of Individual Loci on SWD Incidence and Length. The standardized effect size (β/σ) shows the direction of effect of each locus on SWD length and incidence. Chromosomes are indicated by alternating gray and white bars, and chromosome numbers are marked along the x-axis. Figure 4. Interaction Plots Representing All Interactions Between Chromosomal Segments (a–b) Each panel shows a representative interaction between two chromosomal regions. All significant interactions are plotted. The left-hand section of each figure shows a cartoon depiction of the interaction. Green arrows between chromosomal regions indicate enhancing interactions, and red arrows indicate suppressive interactions. Green arrows to phenotypes indicate positive main effects, and red arrows indicate negative main effects. The right-hand section of each panel shows the interaction plot for the interaction depicted in the cartoon. The normalized phenotype of each genotype group is shown with [file NIHMS631779-supplement-Supp_FigureS1-S4.pdf]
